# Patterns, trends, and factors influencing hospitalizations for craniosynostosis in Western Australia. A population-based study

**DOI:** 10.1007/s00431-023-04922-4

**Published:** 2023-03-11

**Authors:** Mohammed Junaid, Linda Slack-Smith, Kingsley Wong, Timothy Hewitt, Jenny Bourke, Gareth Baynam, Hanny Calache, Helen Leonard

**Affiliations:** 1 School of Population and Global Health, The University of Western Australia, Clifton Street Building, Clifton Street, Nedlands, 6009 WA Australia; 2 Telethon Kids Institute, The University of Western Australia, Northern Entrance, 15 Hospital Avenue, Nedlands, WA Australia; 3 Department of Plastic and Reconstructive Surgery, Perth Children’s Hospital, Nedlands, WA Australia; 4 Western Australian Register of Developmental Anomalies, Department of Health, Government of Western Australia, Perth, WA Australia; 5 Deakin Health Economics, Institute of Health Transformation, School of Health and Social Development, Faculty of Health, Deakin University, Geelong, VIC Australia; 6 Department of Dentistry and Oral Health, La Trobe Rural Health School, La Trobe University, Bendigo, VIC Australia

**Keywords:** Craniosynostosis, Hospital admissions, Rare diseases, Longitudinal study, Linked data study

## Abstract

Understanding hospital service use among children with a diagnosis of craniosynostosis (CS) is important to improve services and outcomes. This study aimed to describe population-level trends, patterns, and factors influencing hospitalizations for craniosynostosis in Western Australia. Data on live births (1990–2010; *n* = 554,624) including craniosynostosis, episodes of death, demographic, and perinatal factors were identified from the midwives, birth defects, hospitalizations, and death datasets. Information on craniosynostosis and non-craniosynostosis-related admissions, cumulative length of hospital stay (cLoS), intensive care unit, and emergency department–related admissions were extracted from the hospitalization dataset and linked to other data sources. These associations were examined using negative binomial regression presented as annual percent change and associations of hospitalizations by age groups, demographic, and perinatal factors were expressed as incidence rate ratio (IRR). We found an increasing trend in incident hospitalizations but a marginal decline in cLoS for craniosynostosis over the observed study period. Perinatal conditions, feeding difficulties, nervous system anomalies, respiratory, and other infections contributed to majority of infant non-CS-related admissions.Respiratory infections accounted for about twice the number of admissions for individuals with CS (IRRs 1.94–2.34) across all observed age groups. Higher incidence of non-CS hospitalizations was observed among females, with associated anomalies, to families with highest socioeconomic disadvantage and living in remote areas of the state.

*  Conclusion*: Marginal reduction in the cLoS for CS-related admissions observed over the 21-year period are potentially indicative of improved peri-operative care. However, higher incidence of respiratory infection-related admissions for syndromic synostosis is concerning and requires investigation.

**Supplementary Information:**

The online version contains supplementary material available at 10.1007/s00431-023-04922-4.

## Introduction

Children born with craniosynostosis (CS) often require early neurosurgical intervention, ideally between 4 and 13 months of age, with syndromic cases undergoing additional timed craniofacial corrections from childhood into early adulthood [1–3]. Extensive research has been conducted on techniques, costs, complications, and length of stay associated with surgical admissions for CS [4–6]. However, very little is known about the population-level trends and patterns of hospital admissions for craniosynostosis from childhood to early adulthood.

A previous Australian-based population-based study reported a stable trend in hospitalization rates and a marginal decrease in the average length of stay for people with craniosynostosis using the total population separation data from the publicly available Australia-wide National Hospital Morbidity Database [7]. However, these de-identified data were not linked to specific individuals, hence were unable to report on unit-level admissions, patterns of non-surgical admissions for craniosynostosis, provide procedure information for separations, or explain factors influencing admissions [7].

Given the knowledge gaps in the literature regarding the hospital service use for craniosynostosis, it is important to build comprehensive evidence to inform research, clinical and surgical practice, and planning and improve patient care. Hence, this study investigated trends, age-specific patterns, and factors influencing hospital admissions for people with craniosynostosis utilizing the Western Australian linked population data.

## Methods

### Study design and data sources

This retrospective cohort study utilized record-linked deidentified data from four data collections made available by the Western Australian Data Linkage System (WADLS). As a result of this linkage, each individual record is provided with a common unique identification number (linkage key) which was used to link the four data collections to facilitate the analysis.

Cases of craniosynostosis were principally identified from Western Australian Register of Developmental Anomalies (WARDA), with additional cases identified from the Hospital Morbidity Data Collection (HMDC). WARDA is a statutory population-based statewide register of congenital anomalies that identifies eligible cases using multiple sources of ascertainment. It includes structural and functional anomalies diagnosed in stillbirths and livebirths to six years of age [8]. Midwives Notification (MNS) collects information on all births attended by midwives in Western Australia (WA) with a gestational age ≥ 20 weeks or birth weight ≥ 400 g. Information on the Indigenous status, antenatal, and perinatal factors can also be extracted from this legislated surveillance system [9]. The Western Australian Death Registrations records all registered deaths and the associated cause of mortality [10]. The Hospital Morbidity Data Collection (HMDC) is a comprehensive collection of all hospital separations of admitted patients from all public and private hospitals in WA [11].

### Study population

Our overall study population comprised all infants born in WA between 1990 and 2010 (21 years; *n* = 554,624). Among them, all infants born with craniosynostosis were largely identified from WARDA using the BPA-ICD9 codes with diagnostic descriptions for craniosynostosis (75600), Crouzon syndrome (75601), Apert syndrome (75550), Pfeiffer syndrome (75601), Carpenter syndrome (75984), Saethre-Chotzen syndrome (75550), Muenke syndrome (75600), and Baller-Gerold syndrome (75600)]. Some additional cases not identified in WARDA from 1998 onwards were extracted from the separation data of HMDC, provided the individual separation data had both a principal diagnostic code and procedure codes directly corresponding to craniosynostosis (Supplementary Fig. 1). The International Classification of Diseases-10^th^ edition-Australian Modification (ICD-10-AM) diagnostic codes used included Q75.0 (Craniosynostosis), Q75.01 (coronal synostosis), Q75.02 (sagittal synostosis), Q75.03 (metopic synostosis), Q75.04 (multiple suture synostosis), Q75.09 (unclassified synostosis), and Q75.1 (craniofacial dysostosis). The Australian Classification of Health Interventions (ACHI) procedure codes for identifying craniosynostosis included 40115–00, 40118–00 [other procedure for craniosynostosis], and 45785–03 [total cranial vault reconstruction]. Considering the International Classification of Diseases-9^th^ revision system-clinical modification (ICD-9-CM) codes do not specifically identify craniosynostosis, case identification from HMDC was restricted to births following the introduction of the ICD-10-AM system in 1998.

For analysis purposes, only cases of craniosynostosis identified from WARDA could be categorized according to syndromic status as follows: (i) non-syndromic (isolated single/multiple suture synostosis); (ii) syndromic synostosis (presence of additional anomalies).

The comparison cohort included all live-born individuals over the same period but not diagnosed with craniosynostosis. The comparison cohort was extracted from the MNS.

### Outcome

The primary outcome of this study was the number of incident hospitalizations per total person-years. Individual person time under observation was obtained by subtracting either the final date of follow-up (i.e., December 31, 2010) or date of death, whichever occurred earlier, from the date of birth. The value was then summed among the study population to ascertain the total person-years. Hospitalizations in our study were defined as craniosynostosis or non-craniosynostosis-related admissions that included both surgical and non-surgical admissions. All principal diagnoses codes were mapped to the International Classification of Diseases-9^th^ revision system-clinical modification (ICD-9-CM) codes and procedure codes (principal and additional procedure codes) to the Australian Classification of Health Interventions (ACHI) to allow comparisons across the study period.

Craniosynostosis-related admissions (CS-related) considered for this study included surgical and non-surgical admissions that were identified using principal diagnostic codes for craniosynostosis or where relevant principal and additional procedure codes, indicating craniosynostosis repair regardless of principal diagnosis code (Supplementary Table 1). Procedures were broadly grouped into two categories: (i) neurosurgical interventions; (ii) cranio-maxillofacial interventions (Supplementary Table 1).

All admissions not categorized as craniosynostosis-related were broadly considered as non-craniosynostosis (non-CS) related. We specifically considered admissions for ocular, auricular, dental, respiratory infections, other respiratory conditions, and sleep-related disorders reasons separately as these admissions are common or previously reported among children with craniosynostosis [12]. Furthermore, as was done previously [13], we identified post-operative complications after craniosynostosis surgery by different principal diagnostic codes for infection, wound disruption, hemorrhage, dural tear, and cerebrospinal fluid (CSF) leak (Supplementary Table 2).

Secondary outcomes for this study were as follows:

- Length of stay: calculated as the time between admission and discharge. The length of hospital stay was considered as 0.5 days when the admission and discharge occurred on the same day to reflect some period of hospitalization [14]. We reported the cumulative length of stay (cLoS) in this study, which was the total number of admitted days for an individual.
- Frequency of intensive care unit (ICU) admissions: data were extracted from the HMDC and additionally included individual length of stay in intensive care.
- Frequency of emergency department (ED) admissions: this information was collected from the HMDC.

### Covariates

As was done previously [15, 16], information on demographic factors (infant sex, birth year and Indigenous status, remoteness of residence, socioeconomic disadvantage) and perinatal factors (parity, plurality, gestational age, birth weight, and fetal distress, intrauterine growth restriction and percentage of optimal head circumference) for case and comparison cohorts was collected from the MNS.

### Statistical analysis

Descriptive statistics were used to summarize the frequency of admitted individuals (craniosynostosis and non-craniosynostosis-related), intensive care admissions, and emergency admissions. Linear trends in hospitalizations adjusted for age at separation, sex, Indigenous status, remoteness, and socioeconomic disadvantage were estimated using negative binomial regression, and annual percent change (APC) was reported. The APC was calculated by exponentiating the coefficient of the year of birth and then subtracting 1 [17].

Furthermore, we conducted the analysis by type of craniosynostosis (non-syndromic and syndromic) for four age groups. As was done previously [18], the four age groups included infancy (up to 1 year), toddler and preschool (from 1 up to 5 years), primary school (from 5 up to 12 years), adolescence and early adulthood (12 to 21 years). Association of incident hospitalizations per total person-years (all hospitalizations, craniosynostosis and non-craniosynostosis-related) and cumulative length of stay with the type of craniosynostosis adjusted for sex, Indigenous status, remoteness, socioeconomic disadvantage, and birth year were calculated using negative binomial regression and reported as an incidence rate ratio (IRR) and 95% confidence intervals (CI).

A similar modeling approach was used to determine the association between each relevant explanatory variable (demographic and perinatal factors) and hospitalizations (all-cause, craniosynostosis and non-craniosynostosis-related) and IRRs with 95% CIs were reported. All analyses were carried out using Stata 16.0 (Stata Corp, College Station, TX, USA).

As done in our previous work involving missing data, a complete case analysis was also used in this study (Supplementary Table 3) as we assumed the mechanism of missingness to be missing at random [15, 16].

This study was performed in line with the principles of the Declaration of Helsinki. The protocol was approved by the Human Research Ethics Committee of the WA Department of Health (HREC#2011/64), Western Australian Aboriginal Health Ethics Committee (HREC#2015/613) and The University of Western Australia (RA/4/20/5843).

## Results

As presented in Table 1, nearly all (96.9%) individuals with craniosynostosis were hospitalized. Ten individuals had no admission records during the study period. Furthermore, under 5% (4.3%; 14/322) of CS individuals, all of whom had syndromic conditions, died at any given point during the study period. Mean incident hospitalizations was lowest for non-syndromic lambdoid synostosis and the highest for Apert syndrome (Table 1).

**Table 1:** Summary and trends of hospitalizations among individuals born with craniosynostosis between 1990 and 2010 in Western Australia

**Total number of individuals identified with craniosynostosis and their subtypes (*n*)**	**No. individuals admitted (*n*, %)**	**No. individuals with CS-related admissions** **(*n*, %)**^**e**^	**No. individuals with non-CS-related admissions** **(*n*, %)**^**e**^	**No. individuals with ICU-related admissions** **(*n*, %)**^**e**^	**No. individuals with emergency-related admissions** **(*n*, %)**^**e**^	**Mean hospitalizations per 1000 py (95% CI)**	**Annual percent change (95% CI)**^**a**^			**Incidence rate ratio, (95% CI)**^**bc**^
							**All-cause hospitalizations**	**Craniosynostosis-related admissions**	**Non-craniosynostosis-related admissions**	
Overall (322)^f^	312 (97)	269 (86)	268 (86)	140 (45)	185 (59)	521.68 (497.44, 546.79)	8.25 (6.55, 9.97)	13.30 (11.29, 15.35)	6.63 (5.45, 7.84)	2.04 (1.94, 2.14)
Comparison cohort (554,302)	350,531 (63)	2,611 (7)	350,285 (99)	30,353 (9)	236,228 (67)	227.41 (226.93, 227.90)	10.27 (9.88, 10.66)	4.93 (3.71, 6.17)	10.26 (9.87, 10.65)	
Non-syndromic (188)^gd^	183 (97)	164 (89)	152 (83)	71 (39)	101 (55)	352.27 (327.21, 378.74)	8.44 (6.64, 10.27)	12.88 (10.19, 15.63)	6.89 (4.94, 8.67)	1.42 (1.31, 1.53)
Sagittal synostosis (96)	95 (99)	92 (97)	78 (82)	42 (44)	52 (55)	386.23 (348.37, 427.09)				
Coronal synostosis (24)	23 (96)	20 (87)	18 (78)	9 (39)	10 (43)	296.98 (236.54, 368.15)				
Metopic synostosis (33)	31 (94)	24 (73)	26 (84)	14 (45)	19 (61)	405.65 (336.60, 484.70)				
Lambdoid synostosis (24)	23 (96)	19 (79)	21 (91)	≤ 5	13 (56)	217.00 (173.57, 267.99)				
Multiple suture synostosis (10)	10 (100)	9 (90)	8 (80)	≤ 5	6 (60)	522.96 (391.73, 684.04)				
Syndromic (119)^g^	114 (96)	90 (76)	103 (90)	47 (37)	79 (69)	796.62 (746.50, 849.21)	8.20 (5.54, 8.96)	11.32 (8.91, 13.78)	6.96 (5.36, 8.59)	3.09 (2.89, 3.30)
**Contributing syndromes**										
Crouzon syndrome (18)	16 (89)	12 (67)	16 (89)	≤ 5	12 (67)	834.86 (713.39, 971.08)				
Apert syndrome (≤ 5)	≤ 5	≤ 5	≤ 5	≤ 5	≤ 5	1739.43 (1313.95, 2258.80)				
Pfeiffer syndrome (≤ 5)	≤ 5	≤ 5	≤ 5	0	≤ 5	416.56 (242.66, 666.95)				
Muenke syndrome (7)	7 (100)	6 (86)	≤ 5	6 (86)	≤ 5	1598.88 (1157.11, 2153.67)				
Saethre-Chotzen syndrome (≤ 5)	≤ 5	≤ 5	≤ 5	≤ 5	≤ 5	607.62 (396.92, 890.30)				

*CS* craniosynostosis, *ICU* intensive care unit, *py* person-years, *CI* confidence interval

^a^Adjusted for sex, Indigenous status, remoteness, socioeconomic disadvantage and age at hospital separation

^b^Adjusted for birth year, sex, Indigenous status, remoteness, socioeconomic disadvantage, and age at hospital separation

^c^Reference group—comparison cohort

^d^Includes sagittal, coronal, metopic, lambdoid, multiple suture and unspecified craniosynostosis

^e^Reflects proportion of individual hospitalizations to those admitted

^f^Total of 307 cases identified from Western Australian Register of Developmental Anomalies (WARDA) and 15 cases from Hospital Morbidity Data Collection

^g^Includes cases identified in WARDA alone

The median cumulative length of stay (cLoS) was lowest for those with non-syndromic coronal synostosis and highest for those with Apert syndrome. Furthermore, about three-fifths of the admitted individuals with CS had an emergency admission, and more than two-fifths required intensive care (Supplementary Table 4). While the median cumulative length of stay for individuals with CS in intensive care was only one day (IQR 1,16), children with Crouzon syndrome, on average, spent a total of 53 days (IQR 37, 58) in intensive care (Supplementary Table 4).

### Trends of hospital admissions and cumulative length of stay

A steady increase in individual hospitalizations for both case and comparison cohort, including all-cause, CS, and non-CS-related admissions was observed over the study period. The incident hospitalizations were twice as high for craniosynostosis [IRR 2.04 (95% CI 1.94, 2.14)], more pronounced for syndromic CS, than those born without this condition (Table 1).

We observed a marginal decline in the trend for the cumulative length of stay for all-cause and CS-related hospitalizations [APC − 1.10 (95% CI − 3.23, 1.08)] prominently for non-syndromic CS but not for non-craniosynostosis-related admissions. Individuals with craniosynostosis spent three and a half times more total days in hospital for any cause [IRR 3.56 (95% CI 3.07, 4.12] than those without (Supplementary Table 4).

### Patterns of hospital admissions and cumulative length of stay by age group

While the frequency of all-cause hospitalizations, CS-related and non-CS-related, ICU, and ED hospitalizations declined with increasing age, non-CS-related admissions contributed to a larger proportion of all admissions for ages after infancy (Table 2). This declining pattern was more pronounced for non-syndromic CS. Furthermore, we observed limited instances of post-operative complications, most of which occurred during the first 5 years of life (Table 2).

**Table 2:** Patterns of hospitalizations for children born between 1990 and 2010 by type of craniosynostosis and age group in Western Australia

**Age**	**Explanatory variables**	**Craniosynostosis**		
		**Overall**	**Non-syndromic**	**Syndromic**
Under 1 year	*n* infants	322	188	119
	Not hospitalized	26	12	14
	*n* admitted (%)	296 (92)	176 (94)	105 (88)
	Mean hospitalizations in 1000 py	2616.26 (2439.59, 2802.34)	2173.32 (1966.03, 2396.51)	3315.15 (2985.48, 3671.27)
	**Incidence rate ratio (95% CI)**^**a**^	**4.28 (3.81, 4.82)**	**3.41 (2.91, 4.00)**	**5.60 (4.64, 6.77)**
	*n* (%) of individuals with craniosynostosis-related admissions^b^	229 (77)	148 (84)	69 (66)
	*n* (%) of individuals with non-craniosynostosis-related admissions^b^	224 (76)	125 (71)	89 (85)
	*n* (%) of individuals with ICU admissions	120 (40)	68 (39)	42 (40)
	*n* (%) of individuals with emergency admissions	138 (47)	67 (38)	66 (63)
	*n* (%) of post-operative complications	7 (2.5)	≤ 5	≤ 5
One up to 5 years	*n* children	303	182	108
	Not hospitalized	134	94	35
	*n* admitted (%)	169 (56)	88 (48)	73 (68)
	Mean hospitalizations in 1000 py	493.66 (452.78, 537.23)	270.26 (232.12, 312.88)	893.78 (801.56, 943.71)
	**Incidence rate ratio (95% CI)**^**a**^	**3.22 (2.66, 3.90)**	**1.54 (1.18, 2.02)**	**6.38 (4.71, 8.64)**
	*n* (%) of individuals with craniosynostosis-related admissions^b^	62 (37)	26 (30)	33 (45)
	*n* (%) of individuals with non-craniosynostosis-related admissions^b^	152 (90)	80 (91)	66 (90)
	*n* (%) of individuals with ICU admissions	27 (16)	9 (10)	15 (20)
	*n* (%) of individuals with emergency admissions	91 (54)	50 (57)	40 (55)
	*n* (%) of post-operative complications	7 (4)	≤ 5	≤ 5
Five up to 12 years	*n* children	234	146	81
	Not hospitalized	111	84	22
	*n* admitted (%)	123 (52.6)	62 (42)	59 (73)
	Mean hospitalizations in 1000 py	216.08 (191.59, 242.83)	134.16 (110.26, 161.69)	350.97 (299.31, 408.99)
	**Incidence rate ratio (95% CI)**^**a**^	**2.61 (2.02, 3.36)**	**1.60 (1.13, 2.26)**	**4.31 (2.87, 6.47)**
	*n* (%) of individuals with craniosynostosis-related admissions^b^	18 (15)	6 (9.7)	11 (19)
	*n* (%) of individuals with non-craniosynostosis-related admissions^b^	117 (95)	60 (97)	55 (93)
	*n* (%) of individuals with ICU admissions	7 (5.8)	≤ 5	≤ 5
	*n* (%) of individuals with emergency admissions	52 (42)	30 (48)	21 (36)
	*n* (%) of post-operative complications	≤ 5	≤ 5	≤ 5
12 to 21 years	*n* children	143	89	54
	Not hospitalized	99	68	31
	*n* admitted (%)	44 (31)	21 (24)	23 (43)
	Mean hospitalizations in 1000 py	168.40 (137.87, 203.67)	99.83 (71.64, 135.43)	297.11 (229.31, 378.70)
	**Incidence rate ratio (95% CI)**^**a**^	**1.60 (1.13, 2.26)**	**0.96 (0.60, 1.53)**	**2.86 (1.67, 4.88)**
	*n* (%) of individuals with craniosynostosis-related admissions^b^	≤ 5	0	≤ 5
	*n* (%) of individuals with non-craniosynostosis-related admissions^b^	43 (98)	21 (100)	22 (96)
	*n* (%) of individuals with ICU admissions	≤ 5	≤ 5	≤ 5
	*n* (%) of individuals with emergency admissions	21 (48)	12 (57)	9 (39)
	*n* (%) of post-operative complications	0	0	0

*ICU* intensive care unit, *py* person-years, *CI* confidence interval

^a^Incidence rate ratio report differences in individual all-cause hospitalizations between case and comparison cohort (reference group)

^b^Reflects proportion of individual hospitalizations to those admitted

With respect to surgical admissions, half [50.0%] of children with CS aged up to 5 years undergoing neurosurgical interventions required intensive care. The requirement of intensive care after cranio-maxillofacial interventions was proportionally about 30% higher than neurosurgical admissions for the same age group (Supplementary Table 5).

**Fig. 1:**
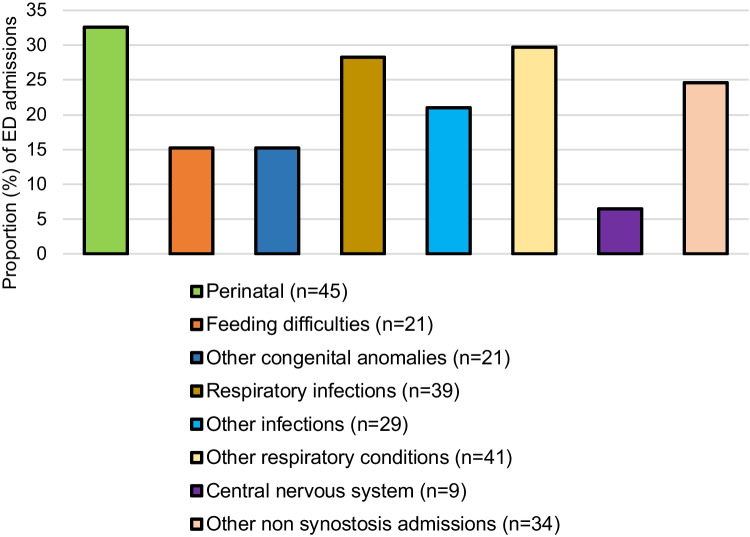
Proportion of non-craniosynostosis admissions^ with an emergency department (ED) admission (*n* = 138) across all ages between 1990 and 2010 in Western Australia. A single circumflex accent (^) indicates a proportion of cases not mutually exclusive

As presented in Table 3, perinatal conditions, other congenital anomalies, and feeding difficulties contributed to a large share of infant admissions, among other non-CS-related reasons. Respiratory infections contributed to about twice the number of hospital admissions for children with CS, especially syndromic CS, for all observed age groups compared to those without (Table 3). Furthermore, males with non-syndromic CS had higher rates of hospitalizations for respiratory infections than females which was contrary to syndromic CS were females had higher hospitalization rates than males (Supplementary Fig. 2). Hospital admissions for oral health–related reasons were also nearly three times higher among children with CS aged between 5 and 12 years relative to those without CS. We also observed that nearly three-fifths of all non-synostosis admissions were recorded as emergency admissions which were even higher for syndromic CS (Table 3). Furthermore, among non-craniosynostosis admissions, perinatal, other respiratory conditions (including asthma), and respiratory infections contributed to a large share of emergency admissions across age groups (Fig. 1).

**Table 3:** Patterns of non-craniosynostosis-related admissions between 1990 and 2010 by type of craniosynostosis and age group in Western Australia

**Age**	**Explanatory variables**	**Craniosynostosis **					
			** Overall**		**Non-syndromic**		** Syndromic**
Up to 1 year	Number of admitted infants		214		125		89
	Cumulative length of stay [median; IQR]		10 (6,18)		9 (5,13)		15 (8,30)
	*n* (%) of individuals with ICU admissions		33 (15)		13 (10)		20 (22)
	*n* (%) of individuals with emergency admissions		126 (59)		63 (50)		63 (71)
	**Principal diagnosis**	***n*** **(%)**	**IRR (95% CI)**	***n* (%)**	**IRR (95% CI)**	***n*** **(%)**	**IRR (95% CI)**
	Perinatal conditions	83 (39)	1.58 (1.24, 2.00)	40 (32)	1.14 (0.81, 1.60)	43 (48)	2.32 (1.66, 3.24)
	Other congenital anomalies^a^	55 (26)	10.81 (5.62, 20.82)	19 (15)	3.46 (1.48, 8.11)	36 (40)	27.21 (9.65, 76.74)
	Feeding difficulties	40 (19)	1.61 (1.20, 2.15)	11 (8.8)	1.27 (0.70, 2.30)	27 (30)	5.95 (4.21, 8.42)
	Eye	≤ 5	–	≤ 5	–	≤ 5	–
	Ear	≤ 5	–	≤ 5	–	≤ 5	–
	Nervous system	17 (7.9)	5.69 (2.59, 12.50)	≤ 5	–	14 (16)	13.08 (4.16, 41.06)
	Sleep disorders	6 (2.8)	1.75 (0.62, 4.99)	≤ 5	–	≤ 5	–
	Respiratory infections	34 (16)	2.34 (1.45, 3.44)	12 (9.6)	1.12 (0.57, 2.20)	22 (25)	4.20 (2.29, 7.70)
	Other infections	16 (7.5)	2.10 (1.20, 3.61)	12 (9.6)	2.72 (1.44, 5.15)	≤ 5	–
	Other respiratory conditions	≤ 5	–	0	–	≤ 5	–
From 1 up to 5 years	Number of admitted children	146		80		66	
	Cumulative length of stay [median; IQR]	3 (1,8)		2 (1,6)		5 (2,14)	
	*n* (%) of individuals with ICU admissions	8 (5.5)		2 (2.5)		6 (9.1)	
	*n* (%) of individuals with emergency admissions	89 (61)		50 (62)		39 (59)	
	**Principal diagnosis**	***n*** **(%)**	**IRR (95% CI)**	***n*** **(%)**	**IRR (95% CI)**	***n*** **(%)**	**IRR (95% CI)**
	Perinatal conditions	≤ 5	–	0	–	≤ 5	–
	Other congenital anomalies^a^	25 (17)	6.25 (2.99, 13.06)	6 (7.5)	1.56 (0.49, 4.97)	19 (29)	14.67 (4.77, 45.05)
	Feeding difficulties	≤ 5	–	0	–	≤ 5	–
	Eye	13 (8.9)	3.86 (1.55, 9.64)	≤ 5	–	8 (12)	6.21 (1.56, 24.78)
	Ear	34 (23)	2.94 (1.89, 4.56)	12 (15)	1.10 (0.55, 2.22)	22 (33)	6.19 (3.25, 11.77)
	Oral health related	12 (8.2)	1.19 (0.62, 2.26)	6 (7)	0.84 (0.32, 2.12)	6 (9.0)	1.83 (0.75, 4.49)
	Nervous system	11 (7.5)	2.53 (0.90, 7.11)	≤ 5	–	10 (15)	7.17 (1.65, 31.09)
	Sleep disorders	≤ 5	–	0	–	≤ 5	–
	Respiratory infections	46 (31)	2.04 (1.44, 2.90)	23 (29)	1.31 (0.79, 2.16)	23 (35)	3.34 (2.01, 5.56)
	Other infections	25 (17)	2.21 (1.44, 3.39)	15 (19)	1.85 (1.05, 3.27)	10 (15)	2.84 (1.47, 5.48)
	Other respiratory conditions	12 (8.2)	1.80 (0.88, 3.70)	≤ 5	–	8 (12)	4.14 (1.43, 12.01)
From 5 up to 12 years	Number of admitted children		115		60		55
	Cumulative length of stay [median; IQR]		2 (1,4)		2 (1,3.5)		2 (1,5)
	*n* (%) of individuals with ICU admissions		≤ 5		≤ 5		≤ 5
	*n* (%) of individuals with emergency admissions		51 (44)		30 (50)		21 (38)
	**Principal diagnosis**	***n*** **(%)**	**IRR (95% CI)**	***n*** **(%)**	**IRR (95% CI)**	***n*** **(%)**	**IRR (95% CI)**
	Other congenital anomalies^a^	16 (14)	6.53 (2.25, 18.92)	≤ 5	–	12 (22)	11.96 (2.26, 63.21)
	Eye	17 (15)	10.87 (3.29, 35.99)	6 (10)	6.27 (1.29, 30.57)	11 (20)	19.40 (2.82, 133.59)
	Ear	21 (18)	2.78 (1.37, 5.62)	≤ 5	–	17 (31)	6.91 (2.39, 19.97)
	Oral health related	28 (24)	2.76 (1.75, 4.38)	8 (13)	1.21 (0.56, 2.62)	20 (36)	5.64 (3.00, 10.60)
	Nervous system	9 (7.8)	2.92 (0.93, 9.13)	≤ 5	–	≤ 5	–
	Sleep disorders	≤ 5	–	≤ 5	–	≤ 5	–
	Respiratory infections	22 (19)	2.05 (1.32, 3.18)	15 (25)	1.94 (1.11, 3.40)	7 (13)	2.24 (1.10, 4.56)
	Other infections	≤ 5	–	≤ 5	–	≤ 5	–
	Other respiratory conditions	≤ 5	–	≤ 5	–	≤ 5	–
From 12 to 21 years	Number of admitted individuals	43		21		22	
	Cumulative length of stay [median; IQR]	2 (1,3)		2 (1,2)		2.5 (1,7)	
	*n* (%) of individuals with ICU admissions	≤ 5		≤ 5		≤ 5	
	*n* (%) of individuals with emergency admissions	21 (49)		12 (57)		9 (41)	
	**Principal diagnosis**	***n*** **(%)**	**IRR (95% CI)**	***n*** **(%)**	**IRR (95% CI)**	***n*** **(%)**	**IRR (95% CI)**
	Other congenital anomalies^a^	≤ 5	–	≤ 5	–	≤ 5	–
	Eye	≤ 5	–	0	–	≤ 5	–
	Ear	≤ 5	–	0	–	≤ 5	–
	Oral health related	10 (23)	1.13 (0.61, 2.11)	≤ 5	–	7 (32)	2.53 (1.21, 5.32)
	Nervous system	≤ 5	–	≤ 5	–	≤ 5	–
	Sleep disorders	≤ 5	–	≤ 5	–	≤ 5	–
	Respiratory infections	8 (19)	1.94 (0.81, 4.67)	≤ 5	–	≤ 5	–
	Other infections	≤ 5	–	0	–	≤ 5	–
	Other respiratory conditions	≤ 5	–	0	–	≤ 5	–

*ICU* intensive care unit, *IQR* interquartile range, *N* number of cases, *IRR* incidence rate ratio; *CI* confidence interval

^a^Includes major and minor congenital anomalies

### Factors influencing hospital admission patterns

All-cause hospitalizations for females with craniosynostosis were 1.46 times (95% CI 1.31, 1.61) higher than for males (Table 4). Furthermore, the most socioeconomically disadvantaged quintile had a 32% greater increase in all-cause hospitalizations [IRR 1.32 95% CI 1.13, 1.55], especially non-CS admissions, than those in the highest quintile (Table 4). Being born preterm increased the risk of both synostosis and non-synostosis-related admissions relative to those born at term. Similarly, low birth weight, greater intrauterine growth restriction, and presence of fetal distress increased the incidence of hospitalizations, especially non-synostosis-related, for individuals with CS, more so for those with syndromic conditions (Table 4).

**Table 4:** Adjusted incidence rate ratios (IRR) and 95% CIs of demographic and perinatal factors for patterns of hospitalizations by type of craniosynostosis in Western Australia

**Explanatory variables**	**Craniosynostosis (IRR, 95% CI)**								
	**Overall**			**Non-syndromic**			**Syndromic**		
	**All-cause hospitalizations**	**CS-related**	**Non-CS-related**	**All-cause hospitalizations**	**CS-related**	**Non-CS-related**	**All-cause hospitalizations**	**CS-related**	**Non-CS-related**
**Gender**									
Female	1.46	1.14	1.59	0.91	0.87	1.02	1.99	1.62	2.02
	(1.31, 1.61)^*^	(0.94, 1.40)	(1.41, 1.09)	(0.77, 1.07)	(0.65, 1.16)	(0.82, 1.25)	(1.72, 2.30)	(1.18, 2.24)	(1.91, 2.38)
Male	Ref	Ref	Ref	Ref	Ref	Ref	Ref	Ref	Ref
**Indigenous status**									
Non-Indigenous	Ref	Ref	Ref	Ref	Ref	Ref	Ref	Ref	Ref
Indigenous	0.91	1.07	0.99	1.45	0.98	1.69	0.4	1.31	0.39
	(0.70, 1.18)	(0.54, 2.09)	(0.74, 1.31)	(1.02, 2.06)	(0.30, 3.20)	(1.16, 2.46)	(0.25, 0.62)	(0.44, 3.86)	(0.23, 0.66)
**Accessibility and remoteness index for Australia (ARIA) at birth**									
Major cities	Ref	Ref	Ref	Ref	Ref	Ref	Ref	Ref	Ref
Outer regional/inner regional	0.92	0.93	0.89	0.94	1.03	0.85	1.14	0.9	1.2
	(0.79, 1.08)	(0.70, 1.24)	(0.74, 1.07)	(0.76, 1.16)	(0.72, 1.47)	(0.65, 1.10)	(0.89, 1.45)	(0.43, 1.85)	(0.92, 1.58)
Remote/very remote	1.11	1.11	0.98	1.15	1.09	1.05	1.56	0.95	1.51
	(0.88, 1.40)	(0.73, 1.69)	(0.74, 1.30)	(0.76, 1.73)	(0.56, 2.15)	(0.62, 1.79)	(1.11, 2.17)	(0.36, 2.48)	(1.01, 2.27)
**Index of relative socioeconomic disadvantage (IRSD) quintiles at birth (Q)**									
Q1 (≤ 20%)	1.32	0.96	1.32	1.9	1.13	1.96	1.15	0.69	1.3
	(1.13, 1.55)^*^	(0.69, 1.37)	(1.10, 1.58)^*^	(1.43, 2.52)	(0.71, 1.79)	(1.35, 2.83)	(0.94, 1.41)	(0.32, 1.49)	(1.04, 1.62)
Q2 (21–40%)	0.89	0.89	0.88	1.72	1.04	1.92	0.73	0.85	0.72
	(0.75, 1.05)	(0.65, 1.23)	(0.73, 1.08)	(1.30, 2.28)	(0.66, 1.63)	(1.32, 2.77)	(0.57, 0.92)	(0.42, 1.73)	(0.55, 0.94)
Q3 (41–60%)	0.92	0.94	0.81	1.62	1.17	1.44	0.66	0.78	0.65
	(0.78, 1.09)	(0.68, 1.28)	(0.67, 0.98)	(1.20, 2.18)	(0.74, 1.86)	(0.97, 2.14)	(0.53, 0.81)	(0.38, 1.64)	(0.52, 0.83)
Q4 (61–80%)	0.81	1.02	0.68	1.56	1.14	1.52	0.64	0.86	0.58
	(0.69, 0.96)^*^	(0.76, 1.37)	(0.56, 0.83)	(1.17, 2.07)	(0.74, 1.76)	(1.04, 2.22)	(0.51, 0.80)	(0.44, 1.69)	(0.45, 0.76)
Q5 (> 80%)	Ref	Ref	Ref	Ref	Ref	Ref	Ref	Ref	Ref
***Perinatal factors***									
**Gestation in weeks**									
Preterm (< 37.00)	1.66	1.41	1.75	1.4	1.02	1.65	1.62	1.74	1.56
	(1.48, 1.87)^*^	(1.10, 1.81)^*^	(1.53, 2.00)^*^	(1.15, 1.70)	(0.67, 1.54)	(1.31, 2.07)	(1.36, 1.92)	(1.20, 2.53)	(1.28, 1.90)
Term (37.00–41.99)	Ref	Ref	Ref	–	–	–	Ref	Ref	Ref
Post-term (≥ 42.00)	5.76	2.6	–	–	–	–	2.4	2.11	–
	(0.62, 53.83)	(0.16, 41.19)					(0.21, 25.56)	(0.03, 177.69)	
< 2500	1.84	1.43	1.82	1.35	0.87	1.38	2.16	1.64	2.43
	(1.60, 2.12)^*^	(1.04, 1.95)^*^	(1.55, 2.15)^*^	(1.02, 1.79)	(0.49, 1.56)	(0.99, 1.93)	(1.79, 2.61)	(1.07, 2.52)	(1.95, 3.01)
2500–2999	1.4	1.03	1.53	1.4	1.05	1.46	1.51	0.99	1.6
	(1.21, 1.63)^*^	(0.75, 1.40)	(1.28, 1.84)^*^	(1.08, 1.80)	(0.67, 1.66)	(1.06, 2.02)	(1.24, 1.85)	(0.61, 1.58)	(1.27, 1.99)
3000–3499	Ref	Ref	Ref	Ref	Ref	Ref	Ref	Ref	Ref
3500–3999	0.93	0.97	0.77	1.25	0.99	1.08	0.9	1.08	0.8
	(0.80, 1.08)	(0.75, 1.26)	(0.64, 0.93)	(1.00, 1.55)	(0.70, 1.41)	(0.80, 1.44)	(0.71, 1.14)	(0.68, 1.70)	(0.61, 1.05)
> 4000	0.85	0.94	0.72	0.97	0.88	0.86	1	0.98	0.99
	(0.71, 1.03)	(0.67, 1.30)	(0.57, 0.91)	(0.74, 1.27)	(0.57, 1.36)	(0.61, 1.22)	(0.70, 1.44)	(0.45, 2.13)	(0.65, 1.50)
**Proportion of optimal head circumference**^**c**^									
< 95%	0.96	1.22	0.87	0.83	0.97	0.8	1.05	1.23	0.91
	(0.80, 1.14)	(0.88, 1.69)	(0.70, 1.08)	(0.61, 1.14)	(0.56, 1.68)	(0.54, 1.17)	(0.83, 1.34)	(0.76, 1.99)	(0.69, 1.20)
≥ 95 to < 105%	Ref	Ref	Ref	Ref	Ref	Ref	Ref	Ref	Ref
≥ 105%	1.06	1.14	0.97	1.05	0.99	0.98	1.04	1.5	1
	(0.95, 1.20)	(0.92, 1.42)	(0.84, 1.12)	(0.88, 1.26)	(0.72, 1.35)	(0.78, 1.23)	(0.87, 1.23)	(1.03, 2.17)	(0.82, 1.21)
**Proportion of optimal birth weight**									
< 75.00	1.73	1.29	1.78	0.98	1.33	1.1	1.91	1.26	2.11
	(1.41, 2.11)^*^	(0.83, 2.02)	(1.41, 2.23)^*^	(0.64, 1.48)	(0.52, 3.41)	(0.69, 1.75)	(1.46, 2.50)	(0.67, 2.38)	(1.56, 2.85)
≥ 75.00 to < 85.00	1.06	0.85	1.28	0.75	0.8	0.82	1.35	1.01	1.63
	(0.87, 1.30)	(0.54, 1.32)	(1.01, 1.61)^*^	(0.54, 1.06)	(0.44, 1.46)	(0.54, 1.26)	(1.03, 1.75)	(0.42, 2.14)	(1.21, 2.18)
≥ 85.00 to < 95.00	1.07	1.18	1.11	1.04	1.03	1.27	1.1	1.46	1.03
	(0.92, 1.25)	(0.89, 1.57)	(0.93, 1.33)	(0.81, 1.33)	(0.69, 1.55)	(0.93, 1.73)	(0.89, 1.35)	(0.91, 2.34)	(0.81, 1.30)
≥ 95.00 to < 105.00	Ref	Ref	Ref	Ref	Ref	Ref	Ref	Ref	Ref
≥ 105.00 to < 115.00	0.93	0.89	0.92	0.94	0.9	0.95	0.87	0.77	1
	(0.79, 1.11)	(0.64, 1.24)	(0.75, 1.12)	(0.74, 1.19)	(0.60, 1.35)	(0.71, 1.28)	(0.67, 1.13)	(0.40, 1.51)	(0.75, 1.34)
≥ 115.00 to < 125.00	1.09	1.03	0.99	0.84	0.94	0.76	0.91	1.1	0.86
	(0.88, 1.37)	(0.70, 1.53)	(0.75, 1.30)	(0.61, 1.15)	(0.56, 1.57)	(0.51, 1.15)	(0.60, 1.38)	(0.48, 2.53)	(0.52, 1.41)
≥ 125.00	0.97	0.91	0.97	1.18	0.81	1.29	0.98	1.32	1.12
	(0.81, 1.16)	(0.66, 1.26)	(0.79, 1.20)	(0.91, 1.53)	(0.53, 1.25)	(0.93, 1.79)	(0.75, 1.29)	(0.74, 2.35)	(0.81, 1.54)
**Fetal distress**									
Absent	Ref	Ref	Ref	Ref	Ref	Ref	Ref	Ref	Ref
Present	1.19	1	1.29	0.98	1.11	0.96	1.08	0.71	1.2
	(1.06, 1.32)	(0.80, 1.25)	(1.13, 1.46)^*^	(0.81, 1.18)	(0.82, 1.51)	(0.76, 1.22)	(0.93, 1.27)	(0.49, 1.03)	(1.01, 1.43)

*IRR* incidence rate ratio, *CI* confidence interval, *Ref* reference group

## Discussion

This study provides comprehensive population-based information on the hospitalization burden of craniosynostosis in Western Australia. Our findings indicate an increasing trend in incident hospitalizations for craniosynostosis but a marginal decline in the cumulative length of hospital stay for the observed 21 years. Furthermore, respiratory infections accounted for about twice the number of admissions for individuals with CS across all observed age groups. We also observed a higher incidence of non-CS-related hospitalizations among females, children born preterm, severe intrauterine growth restriction, with syndromic CS, and to families with greatest socioeconomic disadvantage.

There are limited population studies describing patterns of hospitalization for craniosynostosis and other rare craniofacial anomalies. The use of population data to describe the changing trends and age-specific patterns of individual-level hospitalizations is a strength of this study. Furthermore, the ability to link to the midwives registry provided the opportunity to explore the association of demographic and perinatal factors with the incidence of admissions. Considering the inclusivity of this linked data, we were able to avoid any chance of selection bias.

Despite the strengths, there were some limitations to this study. The administrative coding for craniosynostosis in the hospital system is not nuanced, and hence we cannot identify or report individual differences in case severity or treatment protocols followed. However, admissions related to different types of CS and procedure information were better coded with the ICD-10-AM diagnosis and ACHI procedure codes than in the earlier systems of coding using ICD-9-CM and the International Classification of Procedures in Medicine.

The marginal reduction in the cumulative length of stay for CS-related admissions which we observed in our cohort was similar to the findings from the national hospital separation data for craniosynostosis [7]. The observed marginal reduction in the cumulative length of stay in our data could also indicate efficiency and improved quality of hospital care [19]. However, we observed an increasing trend in all-cause, CS and non-CS-related hospitalizations in our cohort despite stable trends observed both for reported birth prevalence in WA and national hospital separation data on craniosynostosis [7, 15]. The increasing trends in hospitalization for craniosynostosis-related reasons may relate to the establishment of a specialized craniofacial unit during the observed time period (1993) [Dr Timothy Hewitt, Perth Children’s Hospital, personal communication, 10.01.2022] and changing treatment practices given the growth of a specialized surgical workforce.

A large proportion of CS-related admissions took place during infancy which is in line with the ideal age for neurosurgical intervention [1]. However, admissions for children aged one to five years in our cohort might relate to multiple issues. These include potential delayed diagnosis, increased waiting times, delayed scheduling of surgery especially for syndromic CS, postsurgical complications, and requirement of continued management for specific rare syndromic conditions [20–22]. We observed a substantial decline in admissions, especially for CS-related reasons for children (especially non-syndromic CS) over the age of five, similar to the pattern observed with national hospital separation data for craniosynostosis [7]. This admission pattern is expected for craniosynostosis, considering most single-suture synostosis have fewer CS-related service contacts with increasing age as a result of better surgical outcomes [23]. However, individuals born with syndromic craniosynostosis have prolonged service contacts for managing additional associated anomalies [22].

We observed higher proportions of ICU admissions and cLoS for cranio-maxillofacial interventions than neurosurgical interventions. For syndromic craniosynostosis, a large majority of patients require airway/midface procedures warranting intensive care admission because of potential airway issues [22]. However, cases undergoing neurosurgical interventions are often extubated the day of surgery, observed overnight in intensive care, and discharged [24]. Furthermore, the length of stay for cranio-maxillofacial intervention is longer, especially for cases with any specific airway issues [25]. Nevertheless, this finding can be confounded by admissions to high dependency units or the presence of a specialist nurse in wards which can potentially reduce the requirement of ICU admissions [26–28].

Respiratory infections contributed to twice as many admissions among individuals with CS and occurred more often in those with syndromic CS across all observed age groups than in those without. While acute lower respiratory infections have been reported to be higher among children with any birth defects, children born with syndromic CS specifically present with midfacial deficiencies and micrognathia, which could increase their susceptibility to respiratory infections [29, 30].

We also observed a higher incidence of hospitalizations, especially for non-CS-related reasons, in females than males, despite the birth prevalence of CS among males being greater than females [15]. Further research is required to explore this sex-related disease severity. It is widely acknowledged that preterm and low birth weight children have higher hospital service contacts, especially during infancy [31]. Considering children with craniosynostosis have a higher chance to be born preterm, with low birth weight, intrauterine growth restrictions, and fetal distress than children born without [15], the higher incidence of hospitalizations among this cohort is largely predictable. While the association between socioeconomic disadvantage and the birth prevalence of craniosynostosis in WA is unclear [15], we found those belonging to families with the highest socioeconomic disadvantage were more frequently admitted, especially for non-craniosynostosis reasons. Previous WA population studies have shown increased hospital service use, especially for respiratory infections among socially disadvantaged children not born with any birth defects [32, 33]. We believe a similar pattern exists for our cohort and requires further investigation.

In the future, from a research standpoint, it will be critical that health system coding is nuanced to accommodate more intricate details related to different craniosynostosis types, associated underlying and comorbid rare diseases, and different surgical techniques.

## Conclusion

Our study provides a longitudinal, population-level description of all individual-level hospitalizations for craniosynostosis in Western Australia and emphasizes the increased hospital service needs for those born with this condition relative to those born without. This hospital service use for individuals with craniosynostosis was higher for females, with greater socioeconomic disadvantage, born preterm, with syndromic conditions, low birth weight, intrauterine growth restriction, and fetal distress. The higher incidence of respiratory infections across age groups for syndromic synostosis is concerning and requires further investigation.

## Supplementary Information

Below is the link to the electronic supplementary material.

Supplementary file1 (DOCX 26 KB)

Supplementary file2 (DOCX 23 KB)

Supplementary file3 (DOCX 46 KB)

Supplementary file4 (DOCX 18 KB)

Supplementary file5 (DOCX 17 KB)

Supplementary file6 (DOCX 21 KB)

Supplementary file7 (DOCX 21 KB)

## Data Availability

Due to the nature of the research [ethical/legal/commercial reasons] supporting data is not available.
