# Supplementary material for: Patterns, trends, and factors influencing hospitalizations for craniosynostosis in Western Australia. A population-based study
Source: Eur J Pediatr. 2023 Mar 11;182(5):2379–92. doi: 10.1007/s00431-023-04922-4 (PMC10175457; doi:10.1007/s00431-023-04922-4)
Supplement: Supplementary file 1 — Supplementary file1 (DOCX 26 KB) [file 431_2023_4922_MOESM1_ESM.docx]

# Supplementary Figure 1. Total number of cases considered for data analysis after excluding missing [socioeconomic disadvantage (IRSD) and remoteness (ARIA)] data.

**Total cohort: n=554,624**

**Total case group (n=322)**

Non-cases with incomplete data (missing IRSD and ARIA data) excluded from multivariable negative binomial regression analysis

**n=29**

Cases with complete data considered for

multivariable negative binomial regression analysis

**n=293**

Non-cases with complete data considered for multivariable negative binomial regression analysis

**n=500,768**

**Total case from WARDA**

**n=307**

**Total case from HMDC**

**n=15**

**Total comparison group (n=554,302)**

Non-cases with incomplete data (missing IRSD and ARIA data) excluded from multivariable negative binomial regression analysis

**n=141,092**

WARDA: Western Australian Register for Developmental Anomalies; HMDC: Hospital Morbidity Data Collection
