# Supplementary material for: Patterns, trends, and factors influencing hospitalizations for craniosynostosis in Western Australia. A population-based study
Source: Eur J Pediatr. 2023 Mar 11;182(5):2379–92. doi: 10.1007/s00431-023-04922-4 (PMC10175457; doi:10.1007/s00431-023-04922-4)
Supplement: Supplementary file 2 — Supplementary file2 (DOCX 23 KB) [file 431_2023_4922_MOESM2_ESM.docx]

Supplementary Figure 2. Mean incident hospitalisations per 1,000 person-years for non-craniosynostosis-related admissions by sex of individuals with craniosynostosis in Western Australia.
