# Supplementary material for: Patterns, trends, and factors influencing hospitalizations for craniosynostosis in Western Australia. A population-based study
Source: Eur J Pediatr. 2023 Mar 11;182(5):2379–92. doi: 10.1007/s00431-023-04922-4 (PMC10175457; doi:10.1007/s00431-023-04922-4)
Supplement: Supplementary file 3 — Supplementary file3 (DOCX 46 KB) [file 431_2023_4922_MOESM3_ESM.docx]

# Supplementary Table 1. ICD-9-CM, ICD-10-AM codes used to identify principal diagnosis and ICPM, ACHI codes used to identify principal/additional procedures for craniosynostosis.

| **Principal diagnosis** | **ICD-9-CM codes** | **ICD-10-AM codes** |
| --- | --- | --- |
| Craniosynostosis | 756.00 | Q75.0 |
| Coronal synostosis | 756.00 | Q75.01 |
| Sagittal synostosis | 756.00 | Q75.02 |
| Trignocephaly (metopic synostosis) | 756.00 | Q75.03 |
| Craniosynostosis of multiple sutures | 756.00 | Q75.04 |
| Pfeiffer syndrome | 756.00 | Q75.05 |
| Clover leaf skull | 756.00 | Q75.06 |
| Other and unspecified craniosynostosis | 756.00 | Q75.09 |
| Craniofacial dyostosis (Crouzon syndrome) | 756.00 | Q75.1 |
| Acrocephalosyndactyly (Apert syndrome) | 756.00 | Q87.0 |
| **Procedures** | **ICPM codes** | **ACHI codes** |
| **Neurosurgical** |  |  |
| Total cranial vault reconstruction | 2.06 | 45785-03 |
| Procedures for craniostenosis | 2.0, 2.01, 2.02, 2.04, 2.05, 2.06, 2.07 | 40115-00, 40118-00 |
| Reconstruction of glenoid fossa, zygomatic arch and temporal bone | 2.06 | 45788-00 |
| **Craniofacial** |  |  |
| Reconstruction of mandible, maxilla, zygoma and orbital cavity | 76.43, 76.46, 16.98 | 45608-00, 45608-01, 45608-02, 45608-03, 45608-04, 45791-00, 52122-00, 52122-01, 52122-02, 52122-03, 90683-00, 45590-00, 45590-01, 45593-00, 45593-02, 45593-03 |
| Reconstruction of other facial bones | 76.46 | 90684-00 |
| Frontal bone advancement | 76.46 | 45782-00, 45782-01,45782-02, 45785-00, 45785-01, 45785-02 |
| Orthognathic surgery | 76.64, 76.66, 76.69, | 45731-00, 45731-01, 45735-00, 45741-00, 45747-00, 45732-00, 45732-01, 45738-00, 45744-00, 45752-00, 45753-00, 45754-00, |
| Cleft lip and palate repair | 27.54, 27.62, 27.63 | 45689-00, 45689-01, 45698-00, 45677-00, 45683-00, 45701-00, 45704-00, 45692-00, 45695-00, 45707-00, 45713-00, 52337-00, 45680-00, 45686-00 |

ICD-9-CM: International Classification of Diseases-9-Clinical Modification; ICD-10-AM: International Classification of Diseases-10-Australian Modification; IPCM: International Classification of Procedures in Medicine; ACHI: Australian Classification of Health Intervention

# Supplementary Table 2. ICD-9-CM, ICD-10-AM codes used to identify post-operative complications.

| **Principal diagnosis** | **ICD-9-CM codes** | **ICD-10-AM codes** |
| --- | --- | --- |
| Wound disruption | 998.3, 998.30, 998.32, 998.33 | T81.3, T81.31 |
| Postoperative infection | 998.5, 998.51, 988.59 | T81.4, T81.41, T81.42 |
| Complications of anesthesia | 995.4 | T88.2, T88.3, T88.4, T88.5 |
| CSF leak and dural tears | 997.0, 997.01, 997.02, 997.09, 996.2, 349.3, 349.31 | G96.0, G97.41 |
| Other complications of surgical and medical care | 998.89, 998.9 | T88.8, T88.9, T81.5, T81.6, T81.7 |

ICD-9-CM: International Classification of Diseases-9-Clinical Modification; ICD-10-AM: International Classification of Diseases-10-Australian Modification; CSF: cerebrospinal fluid

# Supplementary Figure 1. Total number of cases considered for data analysis after excluding missing [socioeconomic disadvantage (IRSD) and remoteness (ARIA)] data.

**Total cohort: n=554,624**

**Total case group (n=322)**

Non-cases with incomplete data (missing IRSD and ARIA data) excluded from multivariable negative binomial regression analysis

**n=29**

Cases with complete data considered for

multivariable negative binomial regression analysis

**n=293**

Non-cases with complete data considered for multivariable negative binomial regression analysis

**n=500,768**

**Total case from WARDA**

**n=307**

**Total case from HMDC**

**n=15**

**Total comparison group (n=554,302)**

Non-cases with incomplete data (missing IRSD and ARIA data) excluded from multivariable negative binomial regression analysis

**n=141,092**

WARDA: Western Australian Register for Developmental Anomalies; HMDC: Hospital Morbidity Data Collection

# Supplementary Figure 2. Mean incident hospitalisations per 1,000 person-years for non-craniosynostosis-related admissions by sex of individuals with craniosynostosis in Western Australia.
