# Supplementary material for: Patterns, trends, and factors influencing hospitalizations for craniosynostosis in Western Australia. A population-based study
Source: Eur J Pediatr. 2023 Mar 11;182(5):2379–92. doi: 10.1007/s00431-023-04922-4 (PMC10175457; doi:10.1007/s00431-023-04922-4)
Supplement: Supplementary file 4 — Supplementary file4 (DOCX 18 KB) [file 431_2023_4922_MOESM4_ESM.docx]

Supplementary Table 2. ICD-9-CM, ICD-10-AM codes used to identify post-operative complications.

| **Principal diagnosis** | **ICD-9-CM codes** | **ICD-10-AM codes** |
| --- | --- | --- |
| Wound disruption | 998.3, 998.30, 998.32, 998.33 | T81.3, T81.31 |
| Postoperative infection | 998.5, 998.51, 988.59 | T81.4, T81.41, T81.42 |
| Complications of anesthesia | 995.4 | T88.2, T88.3, T88.4, T88.5 |
| CSF leak and dural tears | 997.0, 997.01, 997.02, 997.09, 996.2, 349.3, 349.31 | G96.0, G97.41 |
| Other complications of surgical and medical care | 998.89, 998.9 | T88.8, T88.9, T81.5, T81.6, T81.7 |

ICD-9-CM: International Classification of Diseases-9-Clinical Modification; ICD-10-AM: International Classification of Diseases-10-Australian Modification; CSF: cerebrospinal fluid
