# Supplementary material for: Patterns, trends, and factors influencing hospitalizations for craniosynostosis in Western Australia. A population-based study
Source: Eur J Pediatr. 2023 Mar 11;182(5):2379–92. doi: 10.1007/s00431-023-04922-4 (PMC10175457; doi:10.1007/s00431-023-04922-4)
Supplement: Supplementary file 5 — Supplementary file5 (DOCX 17 KB) [file 431_2023_4922_MOESM5_ESM.docx]

Supplementary Table 3: Demographic, and perinatal characteristics for 554,624 live births in WA, including 322 individuals born with craniosynostosis between 1990 and 2010.

| **Explanatory variables** | | **Comparison cohort, n (%)**  **(n=554,302)** | **Craniosynostosis (n=322)** |
| --- | --- | --- | --- |
| **Demographic variables** | |  |  |
| Sex | Female | 270,461 (49) | 112 (35) |
|  | Male | 283,941 (51) | 210 (65) |
| Indigenous status | Non- Indigenous | 517,769 (93) | 309 (96) |
|  | Indigenous | 35,494 (6.4) | 13 (4.0) |
|  | Missing | 1,039 (0.2) | 0 |
| Accessibility and remoteness index for Australia (ARIA) at birth | Major cities | 363,499 (66) | 236 (73) |
|  | Outer regional/ Inner regional | 102,042 (18) | 43 (13) |
|  | Remote/very remote | 37,544 (6.8) | 17 (5.3) |
|  | Missing | 51,217 (9.2) | 26 (8.0) |
| Index of Relative Socioeconomic Disadvantage (IRSD) Quintiles at birth (Q) | Q1 (≤ 20%) | 108,852 (20) | 61 (19) |
|  | Q2 (21-40%) | 107,766 (19) | 61 (19) |
|  | Q3 (41-60%) | 97,549 (18) | 60 (19) |
|  | Q4 (61-80%) | 97,401 (17) | 59 (18) |
|  | Q5(>80%) | 89,388 (16) | 52 (16) |
|  | Missing | 53,346 (9.6) | 29 (9.0) |
| **Perinatal variables** | | | |
| Parity | First | 162,773 (29) | 98 (30) |
|  | Second | 169,861 (31) | 97 (30) |
|  | Third | 106, 038 (19) | 55 (17) |
|  | Fourth or more | 114,585 (21) | 72 (22) |
|  | Missing | 1,045 (0.2) | 0 |
| Plurality | Singleton | 538,283 (97) | 305 (95) |
|  | Twins or more | 16,019 (3) | 17 (5) |
| Gestation in weeks | Preterm (<37.00) | 45,769 (8) | 54 (17) |
|  | Term (37.00 – 41.99) | 501,564 (90) | 267 (83) |
|  | Post term ( ≥42.00) | 6,969 (1.3) | 1 (0.3) |
| Birthweight in grams | <2,500 | 34,334 (6) | 44 (14) |
|  | 2,500 to <3,000 | 87,862 (16) | 47 (15) |
|  | 3,000 to >3,500 | 204,085 (37) | 100 (31) |
|  | 3,500 to <4,000 | 167,885 (30) | 85 (26) |
|  | ≥4,000 | 60,136 (11) | 46 (14) |
| POBW | <75.00 | 12,654 (2) | 19 (5.9) |
|  | ≥ 75.00 - < 85.00 | 45,572 (8) | 22 (6.8) |
|  | ≥ 85.00 - < 95.00 | 118,506 (21) | 71 (22) |
|  | ≥ 95.00 - < 105.00 | 145,454 (26) | 67 (21) |
|  | ≥ 105.00 - <115.00 | 88,700 (16) | 56 (17) |
|  | ≥ 115.00 - < 125.00 | 30,573 (5.5) | 26 (8.0) |
|  | ≥ 125.00 | 112,843 (20) | 61 (19) |
| Fetal distress | Absent | 431,426 (77.8) | 236 (73) |
|  | Present | 122,876 (22) | 86 (27) |

CFA – craniofacial anomalies; POBW – percentage of optimal birthweight
