# Supplementary material for: Patterns, trends, and factors influencing hospitalizations for craniosynostosis in Western Australia. A population-based study
Source: Eur J Pediatr. 2023 Mar 11;182(5):2379–92. doi: 10.1007/s00431-023-04922-4 (PMC10175457; doi:10.1007/s00431-023-04922-4)
Supplement: Supplementary file 6 — Supplementary file6 (DOCX 21 KB) [file 431_2023_4922_MOESM6_ESM.docx]

Supplementary Table 4. Summary and trends of cumulative length of stay among cohort of individuals born with craniosynostosis (CS) between 1990 and 2010 in Western Australia.

| **Total number of individuals identified with CS and their subtypes (n)** | **Cumulative length of stay**  **(Median; IQR)** | **Cumulative length of stay for CS-related admissions**  **(Median; IQR)** | **Cumulative length of stay for non-CS-related admissions**  **(Median; IQR)** | **Cumulative length of stay for all intensive care admissions**  **(Median; IQR)** | **Annual percent change (95% CI)^a^** | | | **Incidence rate ratio**  **(95% CI)^bc^** |
| --- | --- | --- | --- | --- | --- | --- | --- | --- |
|  |  |  |  |  | **All-cause hospitalizations** | **CS-related admissions** | **Non-CS-related admissions** |  |
| Overall (312) | 10  (5, 19) | 6  (4, 10) | 15  (10, 31) | 1  (1, 16) | –1.10  (–3.23, 1.08) | –2.66  (–3.23, –0.60) | 0.38  (–1.91, 2.73) | 3.56  (3.07, 4.12) |
| Non-syndromic (183) | 9  (5, 15) | 6  (4, 8) | 14  (9, 18) | 1  (1, 2) | –2.54  (–4.70, –0.34) | –3.18  (–5.35, –0.95) | –1.75  (–4.15, 0.72) | 1.60  (1.36, 1.88) |
| Sagittal synostosis (92) | 8  (5, 15) | 5  (4, 8) | 15  (12, 23) | 1  (1, 1) |  |  |  |  |
| Coronal synostosis (23) | 8  (5, 12) | 6  (5, 10) | 8  (7, 13) | 23.5  (1, 46) |  |  |  |  |
| Metopic synostosis (31) | 8  (4, 15) | 6  (2, 8) | 13.5  (5, 17) | 1  (1, 3) |  |  |  |  |
| Lambdoid synostosis (23) | 10  (6, 15) | 6  (5, 10) | 13  (9, 15) | 3.5  (1, 40.5) |  |  |  |  |
| Multiple suture synostosis (10) | 16  (7, 18) | 6.5  (6, 11.5) | 18  (16, 49) | – |  |  |  |  |
| Syndromic (114) | 16  (7, 34) | 8  (6, 15) | 22  (10, 46) | 15  (2, 45) | 1.06  (–2.69, 4.96) | –1.16  (–4.50, 2.29) | 2.80  (–1.10, 6.85) | 5.08  (4.16, 6.22) |
| **Contributing syndromes** | |  | |  |  |  |  |  |
| Crouzon syndrome (16) | 17.5  (9, 51.5) | 156  (11, 160) | 16  (7, 37) | 53  (37, 58) |  |  |  |  |
| Apert syndrome (≤5) | 97  (24.5, 177) | 104.5  (15, 194) | 97  (34, 160) | – |  |  |  |  |
| Pfeiffer syndrome (≤5) | 16  (8, 32) | 12  (8, 16) | – | – |  |  |  |  |
| Muenke syndrome (7) | 8  (6, 10) | 6  (5, 8) | 9.5  (8, 230) | 1  (1, 98) |  |  |  |  |
| Saethre-Chotzen syndrome (≤5) | 20.0  (11, 31) | – | 25.5  (20,31) | – |  |  |  |  |

CS: craniosynostosis; IQR: interquartile range; CI: confidence interval

^a^ Adjusted for sex, Indigenous status, remoteness, socioeconomic disadvantage and age at hospital separation

^b^ Adjusted for birth year, sex, Indigenous status, remoteness, socioeconomic disadvantage and age at hospital separation

^c^ Reference group – Comparison cohort^s^
