# Supplementary material for: Patterns, trends, and factors influencing hospitalizations for craniosynostosis in Western Australia. A population-based study
Source: Eur J Pediatr. 2023 Mar 11;182(5):2379–92. doi: 10.1007/s00431-023-04922-4 (PMC10175457; doi:10.1007/s00431-023-04922-4)
Supplement: Supplementary file 7 — Supplementary file7 (DOCX 21 KB) [file 431_2023_4922_MOESM7_ESM.docx]

Supplementary Table 5. Summary of patterns of surgical hospitalization between 1990 and 2010 by type of craniosynostosis and age group in Western Australia.

| **Age** | **Procedure** | **Explanatory variables** | **Craniosynostosis** | | |
| --- | --- | --- | --- | --- | --- |
|  |  |  | **Overall** | **Non-syndromic** | **Syndromic** |
| All ages | Neurosurgical procedures | Total number of individuals hospitalised (n) | 181 | 110 | 56 |
|  |  | cLoS [Median, IQR] | 12 (6, 21) | 9 (5,15) | 21.5 (11.5,50.5) |
|  |  | n (%) of ICU admissions | 94 (52) | 49 (44) | 26 (46) |
|  |  | n (%) of emergency admissions | ≤5 | ≤5 | 0 |
|  | Cranio-maxillofacial procedures | Total number of individuals hospitalised (n) | 53 | 22 | 31 |
|  |  | cLoS [Median, IQR] | 12 (6,21) | 8 (6,14) | 21 (10,34) |
|  |  | n (%) of ICU admissions | 42 (79) | 18 (82) | 24 (77) |
|  |  | n (%) of emergency admissions | ≤5 | 0 | ≤5 |
| Under one year | Neurosurgical procedures | Total number of individuals hospitalised (n) | 148 | 95 | 41 |
|  |  | cLoS [Median, IQR] | 10 (5.5, 19) | 8 (5,14) | 21 (11,28) |
|  |  | n (%) of ICU admissions | 74 (50) | 44 (46) | 18 (44) |
|  |  | n (%) of emergency admissions | 0 | 0 | 0 |
|  | Cranio-maxillofacial procedures | Total number of individuals hospitalised (n) | 37 | 18 | 19 |
|  |  | cLoS [Median, IQR] | 8 (6,15) | 7.5 (6,12) | 11 (6,31) |
|  |  | n (%) of ICU admissions | 31 (84) | 16 (89) | 15 (79) |
|  |  | n (%) of emergency admissions | 0 | 0 | 0 |
| One up to five years | Neurosurgical procedures | Total number of individuals hospitalised (n) | 38 | 16 | 19 |
|  |  | CLoS [Median, IQR] | 18.5 (11, 55) | 14 (7.5, 28) | 46 (19, 78) |
|  |  | n (%) of ICU admissions | 19 (50) | 6 (37) | 9 (47) |
|  |  | n (%) of emergency admissions | ≤5 | ≤5 | 0 |
|  | Cranio-maxillofacial procedures | Total number of individuals hospitalised (n) | 10 | ≤5 | 7 |
|  |  | CLoS [Median, IQR] | 41 (12, 62) | 16 (12,49) | 57 (11,93) |
|  |  | n (%) of ICU admissions | 8 (80) | ≤5 | 6 (86) |
|  |  | n (%) of emergency admissions | ≤5 | 0 | ≤5 |
| 5 to 12 years | Neurosurgical procedures | Total number of individuals hospitalised (n) | ≤5 | 0 | ≤5 |
|  |  | CLoS [Median, IQR] | – | – | – |
|  |  | n (%) of ICU admissions | 0 | 0 | 0 |
|  |  | n (%) of emergency admissions | 0 | 0 | 0 |
|  | Cranio-maxillofacial procedures | Total number of individuals hospitalised (n) | 7 | ≤5 | 6 |
|  |  | CLoS [Median, IQR] | 32 (19, 52) | - | 28 (19,46) |
|  |  | n (%) of ICU admissions | ≤5 | 0 | ≤5 |
|  |  | n (%) of emergency admissions | 0 | 0 | 0 |

cLoS: cumulative length of stay; ICU: intensive care unit; n: number; IQR: interquartile range
